# Biomimetic, Mild Chemical Synthesis of CdTe-GSH Quantum Dots with Improved Biocompatibility

**DOI:** 10.1371/journal.pone.0030741

**Published:** 2012-01-23

**Authors:** José M. Pérez-Donoso, Juan P. Monrás, Denisse Bravo, Adam Aguirre, Andrew F. Quest, Igor O. Osorio-Román, Ricardo F. Aroca, Thomas G. Chasteen, Claudio C. Vásquez

**Affiliations:** 1 Laboratorio de Microbiología Molecular, Departamento de Biología, Facultad de Química y Biología, Universidad de Santiago de Chile, Santiago, Chile; 2 Laboratorio de Bioquímica, Facultad de Ciencias Químicas y Farmacéuticas, Universidad de Chile, Santiago, Chile; 3 Laboratorio de Comunicaciones Celulares, Centro de Estudios Moleculares de la Célula (CEMC), Facultad de Medicina, Universidad de Chile, Santiago, Chile; 4 Departamento de Química Inorgánica, Facultad de Química, Pontificia Universidad Católica de Chile, Santiago, Chile; 5 Materials and Surface Science Group, Faculty of Sciences, University of Windsor, Windsor, Ontario, Canada; 6 Department of Chemistry, Sam Houston State University, Huntsville, Texas, United States of America; RMIT University, Australia

## Abstract

Multiple applications of nanotechnology, especially those involving highly fluorescent nanoparticles (NPs) or quantum dots (QDs) have stimulated the research to develop simple, rapid and environmentally friendly protocols for synthesizing NPs exhibiting novel properties and increased biocompatibility.

In this study, a simple protocol for the chemical synthesis of glutathione (GSH)-capped CdTe QDs (CdTe-GSH) resembling conditions found in biological systems is described. Using only CdCl_2_, K_2_TeO_3_ and GSH, highly fluorescent QDs were obtained under pH, temperature, buffer and oxygen conditions that allow microorganisms growth. These CdTe-GSH NPs displayed similar size, chemical composition, absorbance and fluorescence spectra and quantum yields as QDs synthesized using more complicated and expensive methods.

CdTe QDs were not freely incorporated into eukaryotic cells thus favoring their biocompatibility and potential applications in biomedicine. In addition, NPs entry was facilitated by lipofectamine, resulting in intracellular fluorescence and a slight increase in cell death by necrosis. Toxicity of the as prepared CdTe QDs was lower than that observed with QDs produced by other chemical methods, probably as consequence of decreased levels of Cd^+2^ and higher amounts of GSH.

We present here the simplest, fast and economical method for CdTe QDs synthesis described to date. Also, this biomimetic protocol favors NPs biocompatibility and helps to establish the basis for the development of new, “greener” methods to synthesize cadmium-containing QDs.

## Introduction

CdTe QDs are being intensively used for producing solar panels [1], [2], optoelectronic devices [3] and as fluorescent probes in bioimaging and biosensing [4], [5] among other applications. When compared with organic fluorophores, QDs exhibit important advantages such as i) narrow emission spectra, ii) increased chemical stability, iii) “tunable” spectroscopic properties, iv) photochemical stability, v) high quantum yields and vi) the ability to generate multi-color fluorescence with a single excitation wavelength. Given these characteristics, QDs are mainly used in biomedicine, in studies of bioimaging as fluorescent probes [6], protein trafficking [7] and in the treatment of important diseases like cancer [8]–[10].

Although initially CdTe QDs were produced mostly by organic procedures, several protocols for the aqueous synthesis of these NPs have recently been described [11], [12]. A problem hampering the use of CdTe NPs in biological systems has been their poor biocompatibility [10], [13]–[15]. In this context, aqueous synthesis of CdTe NPs capped with different thiols represents a strategy to promote stability and biocompatibility [15], [16].

To date, protocols for synthesizing thioglycolic acid- [17], glutathione- [18], cysteine- [11] and mercaptosuccinic acid-capped CdTe QDs [19] have been described. These QDs exhibit sizes and quantum yields allowing their use as biological probes when fused to different proteins or antibodies [6], [20], [21]. However, two major problems hindering the widespread use of CdTe QDs fused to thiols still remain. First, CdTe QDs synthesis in aqueous media is cumbersome and protocols still require high temperatures, strong reducing agents, hazardous compounds such as H_2_Te (toxic and flammable gas) or reagents such as NaHTe (highly unstable in the presence of oxygen), which are expensive and require great care and inert atmospheres for operation [22], [23]. Second, although using thiols as capping agents has facilitated CdTe QDs applications, biocompatibility levels remain insufficient and the damage they produce in different organisms, organs or cells has not been defined.

An attractive novel alternative to produce QDs that can bypass the above difficulties is NP biosynthesis. In fact, using microorganisms to produce NPs may permit the generation of more biocompatible CdTe QDs, decreasing both the requirement of dangerous reagents for synthesis and the toxicity of the final products. In the last decade, NPs biosynthesis has displayed some progress, particularly in the case of silver, gold and iron NPs found in magnetotactic bacteria magnetosomes [24]–[26]. The toxicity of biosynthesized NPs is substantially lower and CdTe QDs formed extracellulary by *Escherichia coli* or *Saccharomyces cerevisiae* exhibit increased biocompatibility in HeLa cells when compared to chemically produced CdTe QDs [27], [28]. Nevertheless, QDs biosynthesis is still a poorly studied phenomenon and in particular, intracellular synthesis of CdTe NPs has not been reported to date.

In this work, we developed a green chemical method for synthesizing QDs that defines the minimal conditions required for the *in vitro* synthesis of CdTe QDs, and that represents the basis for the future development of protocols for biosynthesizing QDs *in vivo*. The reported biomimetic method requires substrates as CdCl_2_ and TeO_3_^−2^, whose interaction with bacterial cells has been intensively studied. It also involves the cellular reducing agent GSH and conditions allowing the growth and development of some microbes as temperature, pH and the presence of oxygen, among others.

## Results and Discussion

### Synthesizing and characterizing CdTe QDs

To develop a simple protocol for the aqueous synthesis of CdTe QDs, we decided to use CdCl_2_ and K_2_TeO_3_ as the Cd^2+^ and Te^2−^ sources since the effects that these two compounds have on microorganisms are relatively well known [29]–[32]. Both produce oxidative stress mainly by decreasing non-enzymatic antioxidant defenses as consequence of GSH-mediated Cd^2+^ or Te^4+^ reduction [32]–[34]. Additionally and taking into consideration the redox properties of GSH [35], its abundance in cells (eukaryotes and Gram negative bacteria) and the previously described stabilization of CdSe and CdTe QDs by this tripeptide [17], [18], we decided to test this biological thiol as a reducing and capping agent for the aqueous synthesis of CdTe QDs. As result, a simple protocol requiring only CdCl_2_, K_2_TeO_3_ and GSH was developed.

**Figure 1:**
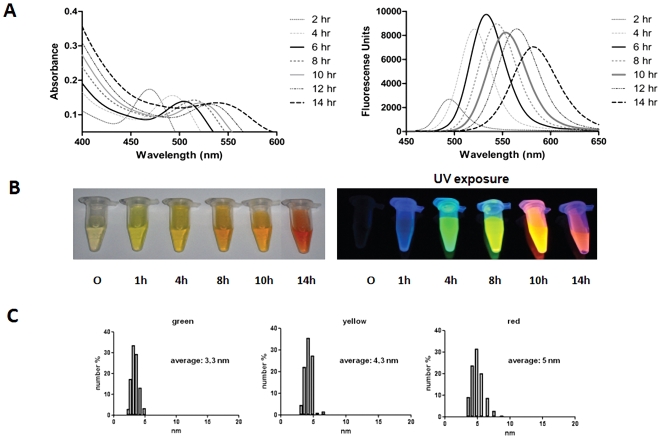
Absorbance and fluorescence emission spectra of CdTe-GSH QDs synthesized at 90°C. **A,** samples were withdrawn at the indicated times. **B,** fluorescence of the as-produced CdTe-GSH QDs at the indicated times of synthesis and excited with UV light at 312 nm. **C,** Dynamic Light Scattering (DLS) analysis of green, yellow and red CdTe-GSH nanoparticles.

A typical reaction at 90°C in citrate buffer, pH 9.0, allowed the generation of highly fluorescent green and red NPs after 2 or 12 h, respectively (Fig. 1). Aliquots withdrawn at the indicated times showed absorbance and fluorescence spectra exhibiting the characteristic CdTe QDs absorption and fluorescence peaks ranging from ∼450–550 nm and ∼500–600 nm, respectively [11], [18], [19] (Fig. 1A). In addition, fractions exposed to UV light (312 nm) were highly fluorescent with an absolute intensity that was proportional to incubation time, shifting from green to yellow and then red after 12 h (Fig. 1B). Absorption and emission peaks were very narrow (with a full bandwidth at half maximum of ∼50 nm), supporting the possibility of using these NPs as cellular probes because of their tunable fluorescence.

Size is an important factor affecting potential biological applications of NPs. CdTe-GSH QDs ranged from 3–6 nm in size with green and red NPs exhibiting average sizes of 3.3 and 5 nm, respectively (Fig. 1C). As expected, yellow NPs exhibited intermediate sizes. A homogeneous, narrow size distribution was observed for all NPs with a distribution range ∼2–3 nm, which favors their use in fluorescent applications (Fig. 1C). Additionally, NPs nanometric size was confirmed by TEM analysis, and most NPs were average sized (4–6 nm), confirming the DLS results previously determined (not shown).

### Elemental composition of the as prepared CdTe-GSH QDs

Energy Dispersive X-ray Spectroscopy (EDS) elemental analysis showed that cadmium, tellurium and other elements such as carbon, sulfur, oxygen and nitrogen were present in QDs. These elements are expected in CdTe-GSH QDs with a GSH-capped CdTe core (Table 1). Interestingly, Te was almost constant in all particles (∼7%) while Cd increased according to NP size. This may reflect thermal decomposition of GSH allowing its loss from NPs surface favoring the deposition of a CdS layer on CdTe-GSH NPs. In this context, most of CdTe-GSH QDs described to date display this kind of CdS layer on their surfaces [22], [36]. Red NPs show increased Cd/Te ratios and less nitrogen and oxygen content, suggesting the formation of the CdS layer on larger CdTe-GSH QDs. To determine that synthesized NPs were actually formed mainly by Cd and Te, and that red NPs display higher concentrations of Cd as those of smaller size (green and yellow), inductively coupled plasma (ICP) experiments were carried out. Results confirmed the presence of Cd and Te at the expected ratio in the synthesized NPs (Table S1). Preliminary surface composition studies of CdTe-GSH NPs using X-ray photoelectron spectroscopy (XPS) confirm this assumption (unpublished data) and indicate that a CdS layer is only present on red NPs.

**Table 1:** EDS elemental analysis of the as-prepared CdTe-GSH NPs. The element per cent in green, yellow and red CdTe-GSH NPs was determined.

Element	NP color		
	green	yellow	red
C	36.57	36.48	35.34
N	15.60	15.50	12.31
O	14.43	11.22	7.10
Na	2.33	1.45	1.02
S	6.02	5.56	5.01
Cd	17.74	22.60	32.24
Te	7.23	7.19	6.98

Since our method uses lower temperatures than other protocols, GSH decomposition and the concomitant production of CdS are decreased, thus favoring NP biocompatibility.

### Infrared spectroscopy

**Figure 2:**
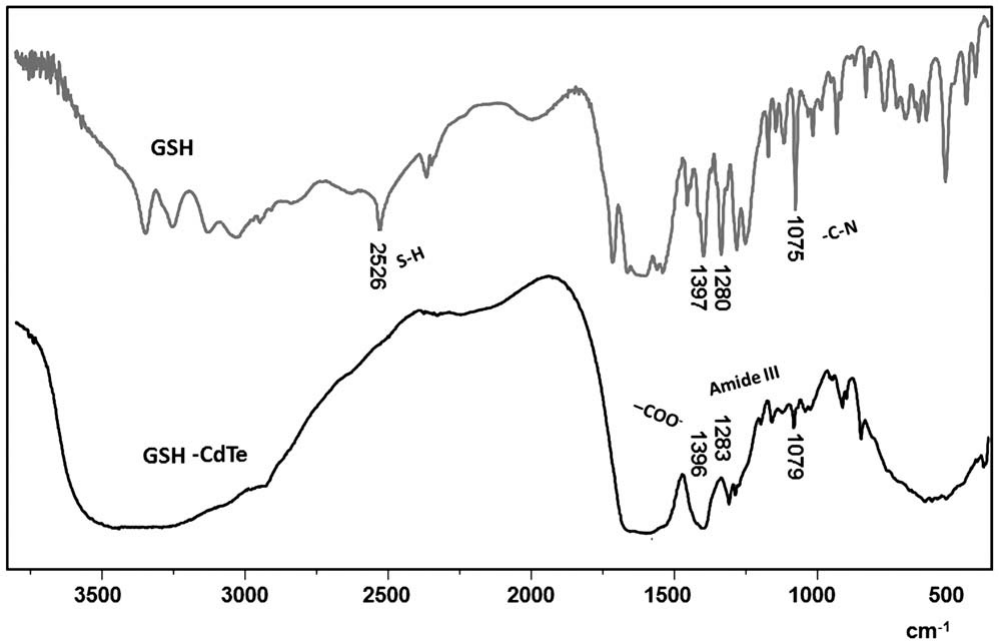
IR spectra of CdTe-GSH NPs and GSH.

To confirm the presence of GSH in QDs, vibrational studies were conducted using CdTe-GSH NPs and GSH as standards (Fig. 2). IR broad absorption bands around 1713–1602 cm^−1^ (symmetric νCOO^−^), 1397 cm^−1^ (asymmetric νCOO^−^), 1713 cm^−1^ (antisymmetric νC = O) and 1280 cm^−1^ (δOH) indicate the presence of a −COOH group, while the band at 1075 cm^−1^ can be assigned to a stretching νC-N. Characteristic νN-H stretching modes observed at 3346 cm^−1^ and 3250 cm^−1^ provide evidence of a -NH_2_ group. The characteristic -SH stretching mode is clearly seen at 2526 cm^−1^. As expected, the IR absorption bands of the main functional groups, -COOH, -NH_2_, and -SH are detected in the neat GSH [37]. In CdTe-GSH NPs, the disappearance of the S-H group vibration at 2526 cm^−1^ (νS-H) is likely the result of covalent bond formation between the thiol and the Cd atom at the CdTe QD surface, suggesting the oxidation of cysteine residues. These IR results are identical to those described for other CdTe-GSH NPs [37], [38]. GSH presence in the as-prepared QDs was also confirmed by Raman spectroscopy (Fig. S1).

### Quantum yield of CdTe-GSH QDs

Regarding fluorescence efficiency, quantum yields (QYs) of green and red CdTe-GSH NPs were about 10 and 30%, respectively. Similar results were obtained with NPs synthesized at 60°C (see below), showing that the best fluorescence yields are associated with larger QDs. These results are similar to those described for other thiol-capped CdTe QDs produced by organometallic chemical methods or other aqueous procedures that use NaBH_4_ as the reducing agent [11], [18], [22], [23]. Higher QYs observed for large aqueous QDs are probably the consequence of thiol ligand decomposition, leading to the above mentioned CdS layer formation on CdTe crystals [36]. This situation may reflect the increase in QYs observed for QDs produced after 10 h at 90°C and is in agreement with Cd and S composition determined for red and green CdTe-GSH QDs (Table 1). The latter could also explain the QY difference observed for NPs of the same color produced at 90°C (∼30%) or 60°C (∼22%). NPs exhibiting QYs ∼80–90% have been reported but it requires complex chemical methods, QDs post-treatment (e.g. photochemical etching and long term illumination) or stabilization with chemical compounds that affect their biological compatibility [14], [22], [39], [40]. X-ray diffraction (XRD) experiments were conducted to confirm the crystalline structure of the as-prepared QDs (not shown). Samples showed some amorphous material which is evidenced by broad bands and low intensity of the diffraction spectrum. This situation could affect NPs optical properties, for example, lower quantum yield. On the other hand, peaks were located between positions for CdTe and CdS crystals (not shown), similarly to other reported QDs. Also, this result may indicate a partial hydrolysis of GSH that could result in a CdS layer deposited on CdTe NPs.

### Effect of pH and temperature on QDs synthesis

**Figure 3:**
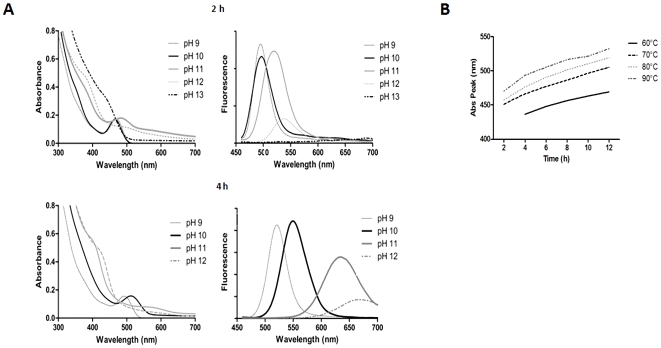
Absorbance and fluorescence emission spectra of CdTe-GSH QDs synthesized at different pHs at 90°C. **A,** above, CdTe-GSH QDs after 2 h; below, same CdTe-GSH QDs after 4 h. Samples prepared at pH 13 showed no fluorescence at 4 h. **B,** Effect of nucleation temperature on CdTe-GSH absorbance peaks measured at different times of synthesis.

The effects of pH and temperature on CdTe QDs synthesis and spectroscopic characteristics were assessed (Figs. 3A and 3B). Highly fluorescent NPs were obtained in a pH range of 9–12 (Fig. 3A). Below pH 8.5 neither fluorescent molecules nor absorption or emission peaks were observed after 48 h. Even though absorption and emission peaks associated with red NPs appear earlier at pHs 11–12, the best spectroscopic results were obtained at pH 9–10 (Fig. 3A). These observations may be explained in terms of the chemical characteristics of GSH, particularly the pK_a_ (8.92) of glutathione's thiol group [41]. In fact, the reducing potential of the GSH/GSSH redox couple becomes more negative as the pH increases [35] and GSH is expected to display maximum reduction potential at pH values near its pK_a_.

The protocol described in this work allows highly fluorescent CdTe-GSH QDs formation in borax-citrate, citrate and phosphate or Tris buffer (not shown). Although QDs were obtained at similar rates, NPs produced in borax-citrate buffer grew faster, with optimal spectroscopic characteristics and no contaminating precipitates (Te°, Cd° or metal oxides).

Since a goal of this study was to develop the basis for producing CdTe NPs by microbes, QDs were synthesized in the presence of microbial culture media. Both rich (LB) and minimal (M9) culture media supported the synthesis of fluorescent CdTe QDs at pH 10–12 after 24 h at 90°C (not shown). QDs synthesis at pH 7.0 was observed in both media only when NaBH_4_ was present. This was intriguing since the NaBH_4_-dependent extracellular production of CdTe NPs by bacterial cells has been previously reported [27], [28]. In this context, we successfully synthesized CdTe NPs *in vitro* under the same conditions described by Bao *et al.* (2010a,b) but in the absence of bacterial cells. Experiments to unveil this apparent controversy are under way in our laboratory.

A direct relationship between QDs synthesis rate and temperature was determined when nucleation temperatures of 60, 70, 80 or 90°C were used (Fig. 3B). In fact, red NPs were obtained after ∼40, 21, 17 and 14 h at these temperatures, respectively. By using the biomimetic procedure, NP synthesis was achieved at in less than two days and after two weeks at 50 and 37°C, respectively.

To test more “biological” temperatures, QDs synthesis was analyzed in more detail at 60°C, a temperature at which several thermophilic microorganisms grow and thus can be potentially used for biosynthesizing CdTe QDs. NP synthesis was slowed down and green or red fluorescent QDs developed only after 24 or 48 h, respectively. Despite this, small QY differences were determined at both temperatures, reinforcing the idea that synthesizing highly fluorescent QDs can be achieved at temperatures that are compatible with bacterial growth.

### CdTe QDs-cell interaction

**Figure 4:**
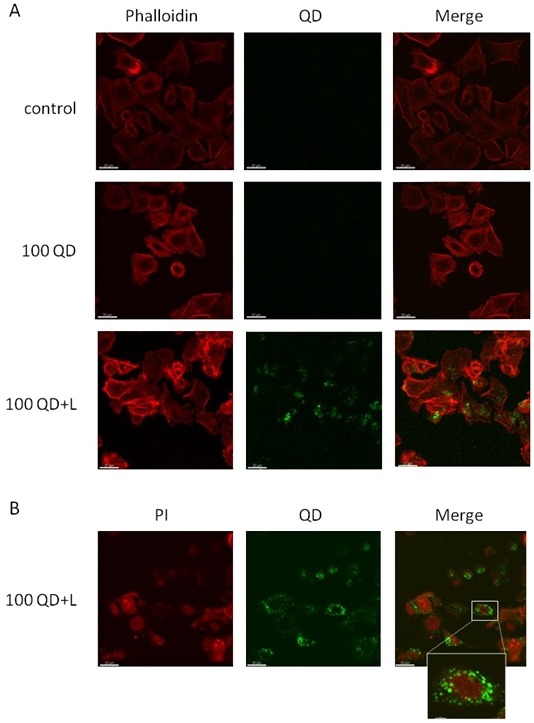
Uptake and intracellular localization of CdTe-GSH QDs by MKN45 cells. **A,** Confocal fluorescence images of MKN45 cells incubated with 100 µg/ml CdTe-GSH QDs in the absence (100 QD) or presence of lipofectamine (100 QD+L). QDs are shown in green and cell cytoplasm was stained by phalloidin (red). **B,** MKN45 cells incubated with 100 µg/ml CdTe-GSH QDs plus lipofectamine (100 QD+L). QDs are shown in green and cell nuclei were stained by PI (red).

To date, various kinds of QDs have been used in cell imaging and biomedicine and it has been reported that their biodistribution and biocompatibility may depend on various factors derived from their physicochemical properties [42]. In this context, MKN45 gastric cancer cells were incubated with 100 µg/ml of CdTe-GSH QDs for 24 h and their cellular distribution and biocompatibility was assessed by fluorescence microscopy. No fluorescence was observed inside MKN45 cells, in contrast with the observation that cysteine- and mercaptopropionic acid-coated CdTe QDs enter human hepatocellular SMMC-7721 [43] and pancreatic carcinoma cells [10], respectively. However, when MKN45 cells were exposed to CdTe NPs in the presence of the cationic liposome lipofectamine, the amount of green fluorescence in the cytoplasm increased in a concentration-dependent manner (Figs. 4A and S2). When MKN45 cells nuclei were stained with propidium iodide (PI), QDs were preferentially detected at the perinuclear region (Fig. 4B). These results show that although not freely incorporated, CdTe-GSH QDs are stable in the cytoplasm making them suitable for cell tracking, labeling and other bioimaging applications. No changes in cell morphology were observed upon incubation with NPs, suggesting that CdTe-GSH QDs do not produce toxic effects at the concentrations tested.

**Figure 5:**
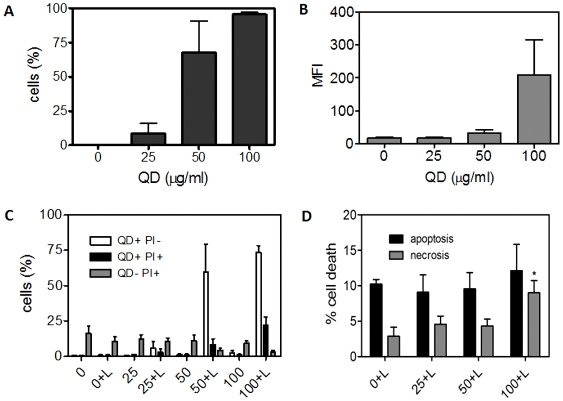
Characterization of QDs incorporation into MKN45 cells by flow cytometry. **A,** Per cent of cells incorporating QDs. **B,** MFI represent the amount of QDs incorporated after incubation with 25, 50 and 100 µg/ml QDs in the presence of lipofectamine. **C,** Viability of MKN45 cells incubated with QDs with or without lipofectamine. The total population of cells (QD^+^PI^−^, QD^+^PI^+^ and QD^−^PI^+^) is referred to as 100%. Numbers indicate concentrations (µg/ml) and L stands for lipofectamine. **D,** Characterization of cell death in MKN45 cells incubated with QDs plus lipofectamine. 100% stands for the total population of QD^+^ cells.

QDs incorporation to MKN45 cells was quantified by flow cytometry after incubating with 25, 50 or 100 µg/ml CdTe-GSH QDs for 24 h in the presence of the same amount of lipofectamine (Fig. 5A). No fluorescent cells were observed in the absence of the liposome (not shown) and in its presence the per cent of cells incorporating QDs increased in proportion to QDs concentration, confirming the results obtained by confocal microscopy (Figs. 4A and S2). The average fluorescence of the cell population was calculated as the median fluorescence intensity value (MFI). MKN45 cells incubated with 100 µg/ml QDs plus lipofectamine exhibited the highest MFI associated with QDs incorporation (Fig. 5B). No significant differences in MFI were observed in cells incubated with 25 or 50 µg/ml QDs as compared to controls, which is in agreement with the low numbers determined in Fig. 5A.

Previous reports indicated that a mechanism underlying CdTe QDs cytotoxicity is related to Cd^2+^ release and the generation of reactive oxygen species (ROS), such as hydrogen peroxide and various hydroperoxide radicals. In turn, ROS can damage proteins, DNA, and lipids thereby leading to severe cell functional impairments and eventually cell death [14]. To demonstrate CdTe-GSH NPs biocompatibility, cell viability of MKN45 cells exposed for 24 h to CdTe QDs was analyzed by flow cytometry, as previously described [44], [45]. Cells were stained with PI to quantify viable (QD^+^ PI^−^) and dead (QD^+^ PI^+^) cells after incubating with QDs either at 25, 50 or 100 µg/ml. Only 10–20% of dead cells were detected in the presence of 50 or 100 µg/ml QDs (Fig. 5C), suggesting that QDs uptake could be responsible for a small increase in cytotoxicity. In the absence of lipofectamine QDs did not induce cell death, regardless the concentration of NPs used, even up to 200 or 400 µg/ml (not shown). The viability of cells exposed to green QDs was very similar in the absence or presence of lipofectamine, at all the concentrations of QDs used (Fig. S3). Basal cell death was close to 18% (Fig. S3 and Fig. 5C), which could result because of cell detachment from the monolayer (see Materials and methods).

### CdTe-GSH mediated cell death

To characterize the type of cell death induced by QDs, i.e. apoptosis or necrosis, fluorescence intensity of the total population of QD^+^ cells was determined [45]. Two populations of PI-permeable (PI^+^) dead cells can be distinguished on the basis of fluorescence intensity, hypodiploid apoptotic cells or necrotic cells with intact DNA [44], [45]. After exposure to CdTe-GSH QDs (100 µg/ml) plus lipofectamine, MKN45 QDs^+^ cells exhibited the same degree of apoptosis as untreated cells (10%) (Fig. 5D), indicating that QDs uptake is not associated with an increased apoptosis. However, a slight increase in necrosis (∼9%) was observed in cells treated with 100 µg/ml CdTe-GSH compared to controls or cells incubated with lower QDs concentrations (Fig. 5D). These results demonstrate that CdTe-GSH QDs display less cytotoxic effects than those previously reported using less biocompatible CdTe QDs [46]–[48].

Significant degrees of toxicity have been described for CdTe QDs under similar conditions to those used here. Experiments carried out with CdTe-MPA or CdTe-cysteamine QDs in rat pheochromocytoma cells (PC12) indicated that these NPs are cytotoxic at concentrations as low as 10 µg/ml [42]. Higher toxicity levels and apoptotic cell death was observed in this cell line with uncoated CdTe at 1 µg/ml [14]. Also, tropism to core histones and histone-rich organelles has been previously reported for CdTe-TGA QDs in macrophage THP1 cells [49]. This observation may explain in part the cellular toxicity of CdTe QDs and why apoptotic cell death is observed in some cases. However, as described here, CdTe-GSH QDs did not localize to the nucleus, the modest levels of death observed were mainly due to necrosis. This result confirms the importance of GSH as a capping agent and indicates that, at least, part of the QDs biocompatibility results from their lack of affinity for particular cell organelles or macromolecules.

Interestingly, mercaptosuccinic acid (MSA)-coated CdTe NPs are less efficiently incorporated into MKN45 cells and generate higher levels of cell death (not shown), indicating that CdTe-GSH QDs produced by the biomimetic method exhibit better biocompatibility than CdTe NPs capped with other thiols as MSA.

In summary and regarding costs, efficiency, low toxicity and because of its simplicity in terms of substrates and conditions required, the method described to produce CdTe QDs is more convenient than other methods published to date. These results favor their potential biological applications and the development of new methods for microorganism-mediated production of CdTe NPs and other similar QDs with biotechnological applications as CdS, CdSe and ZnS, among others.

## Materials and Methods

### Synthesis of CdTe-GSH QDs

A CdCl_2_ (4 mM) solution was prepared in 15 mM borax-citrate buffer, pH 9.0. Then GSH was added (up to 10 mM final) with intense vortexing to avoid the formation of a white precipitate (Cd^0^). After 5 min, tellurite (as K_2_TeO_3_) was added at 1 mM (final concentration) to produce a 4∶10∶1 ratio of CdCl_2_∶ GSH∶ K_2_TeO_3_. At this point the solution turned slightly green as result of CdTe “seeds” formation. QDs nucleation was initiated by raising the temperature up to 90°C. Although these were the basic conditions, different pH values (8.5–13), temperatures (37–90°C), citrate, phosphate, M9 minimal or LB microbiological culture media, were evaluated for synthesizing CdTe-GSH NPs. The reaction could be stopped at any time simply by incubating on ice or at 4°C. To store or to determine production yields, CdTe-GSH NPs can be precipitated with two volumes of ethanol and centrifuged for 20 min at 13,000× *g*. CdTe-GSH QDs in aqueous solution prepared by this method are stable and highly fluorescent for months at room temperature, 4°C or as powder after alcohol precipitation. These aqueous QDs do not precipitate when incubated in various biological buffer solutions (phosphate buffered saline, Tris-HCl, citrate) or in bacterial or cell culture media.

### Spectroscopic characterization

NPs absorption spectra were recorded in a Perkin-Elmer Lambda 11 UV-vis spectrophotometer in a 10-mm quartz cell. NPs fluorescence spectra were recorded in a ISS-PC photoncounting spectrofluorometer. Quantum yields (QY) of fluorescence were assessed by comparing with fluorescein (*φ_f_* = 0.95) in water as described previously [50].

### Nanoparticle composition

The elemental analysis of CdTe-GSH QDs was carried out using a FEI Quanta 200 Environmental Scanning Electron Microscope with an Everhart-Thornley Secondary Electron Detector and a Solid State Backscatter Detector. EDS analysis was performed in an EDAX Si-Li Detector, using Genesis Software.

### IR studies

Infrared transmission spectra of purified NPs were recorded in KBr pellets from 2000 to 400 cm^−1^ on a Nicolet Impact 410 IR spectrophotometer.

### Nanoparticle size determination

Dynamic Light Scattering measurements of aqueous CdTe-GSH QDs were carried out with a Zetasizer nano S90 light scattering system (Malvern Instruments Limited, UK) using a refractive index of 2.6.

### Cell culture

Gastric cancer (MKN45) cell lines were cultured in RPMI medium (GIBCO-BRL, Paisley, Scotland, UK) supplemented with 10% fetal bovine serum and antibiotics (100 U/ml penicillin, 10 µg/ml streptomycin). Cells were incubated at 37°C in a humidified atmosphere with 5% CO_2_.

### Confocal fluorescence imaging

MKN45 cells [51] were cultured on circular cover slips for 24 h and then incubated with QDs in the presence or absence of lipofectamine (24 h). Cells were fixed with PBS-4% paraformaldehyde for 30 min and after washing three times with PBS samples were mounted onto slides with 10% Mowiol–2.5% 1,4-diazobicyclo [2,2,2]octane (DABCO) and visualized with a Carl Zeiss Axiovert-135 M confocal microscope (LSM Microsystems) following excitation at 488 (QDs) or 543 nm (PI). Optical sections obtained for co-localization studies were processed with Imaris software (Bitplane AG, Zurich, Switzerland).

### QDs incorporation into MKN45 cells

After 24 h incubation with QDs or QDs plus lipofectamine, cells were washed twice with PBS, detached with 0,5% trypsin-EDTA and analyzed by flow cytometry (Becton Dickinson, USA). 5,000 events were analyzed in the R1 gated region and size (Forward Scatter - FSC) and granularity parameters were determined (Side Scatter - SSC) excluding cell debris. Results were analyzed using the FCS Express software. The incorporation of QDs was referred as the per cent of positive QDs cells and the amount of QDs incorporated was referred to the mean fluorescence intensity (MFI).

### Viability assays

Cellular viability and cell death were assessed in MKN45 cells exposed for 24 h to QDs or QDs plus lipofectamine. Cells were harvested, washed with FACS Flow buffer (BD, USA), centrifuged at 900 rpm for 5 min, incubated with 10 µg/ml PI and analyzed by flow cytometry. PI negative (not permeable) and positive cells (apoptotic or necrotic) were differentiated according to fluorescence intensity [45]. The same filter was used for detecting green QDs and PI-associated fluorescence (excitation laser line 488 nm), and green and red fluorescence channels were used for QDs and PI, respectively. The emission spectrum maxima for QDs and Pi are 525 and 575 nm, respectively. As control, QDs and PI were read separately before performing a reading with both labels. The extent of apoptosis or necrosis was determined by plotting PI fluorescence vs. the forward scatter parameter, using the FCS-Express software.

## Supporting Information

Figure S1 **Raman vibrational spectra of GSH (a) and CdTe-GSH QDs (b).**
(TIF)

Figure S2 **Confocal fluorescence images of MKN45 cells incubated with 25 or 50 µg/ml CdTe-GSH quantum dots in the presence of lipofectamine (25 QD+L or 50 QD+L).** QDs are shown in green and cell cytoplasm was stained by phalloidin (red).
(TIF)

Figure S3 **Characterization of cell death in MKN45 cells incubated with QDs at the indicated concentrations (mg/ml), with or without lipofectamine.**
(TIF)

Table S1 **Cd and Te content in the as-prepared NPs as determined by ICP.**
(DOCX)

## References

[1] Bang JH, Kamat PV (2009). Quantum dot sensitized solar cells. A tale of two semiconductor nanocrystals: CdSe and CdTe.. ACS Nano.

[2] Kongkanand A, Tvrdy K, Takechi K, Kuno M, Kamat PV (2008). Quantum dot solar cells, tuning photoresponse through size and shape control of CdSe-TiO_2_ architecture.. J Am Chem Soc.

[3] Faraon A, Englund D, Fushman I, Vučković J (2007). Local quantum dot tuning on photonic crystal chips.. Appl Phys Lett.

[4] Hoshino A, Hanaki K, Suzuki K, Yamamoto K (2004). Applications of T-lymphoma labeled with fluorescent quantum dots to cell tracing markers in mouse body.. Biochem Biophys Res Commun.

[5] Han H, Sheng Z, Liang J (2007). Electrogenerated chemiluminescence from thiol-capped CdTe quantum dots and its sensing application in aqueous solution.. Anal Chim Acta.

[6] Hoshino A, Manabe N, Fujioka K, Suzuki K, Yasuhara M (2007). Use of fluorescent quantum dot bioconjugates for cellular imaging of immune cells, cell organelle labeling, and nanomedicine: surface modification regulates biological function, including cytotoxicity.. J Artif Organs.

[7] Muro S, Cui X, Gajewski C, Murciano JC, Muzykantov VR (2003). Slow intracellular trafficking of catalase nanoparticles targeted to ICAM-1 protects endothelial cells from oxidative stress.. Am J Physiol Cell Physiol.

[8] Azzazy HM, Mansour MM, Kazmierczak SC (2007). From diagnostics to therapy: prospects of quantum dots.. Clin Biochem.

[9] Gao X, Cui Y, Levenson RM, Chung LW, Nie S (2004). In vivo cancer targeting and imaging with semiconductor quantum dots.. Nat Biotechnol.

[10] Chang SQ, Dai YD, Kang B, Han W, Mao L (2009). UV-enhanced cytotoxicity of thiol-capped CdTe quantum dots in human pancreatic carcinoma cells.. Toxicol Lett.

[11] Bao H, Wang E, Dong S (2006). One-pot synthesis of CdTe nanocrystals and shape control of luminescent CdTe-cystine nanocomposites.. Small.

[12] He Y, Lu H, Sai L, Lai W, Fan Q (2006). Synthesis of CdTe nanocrystals through program process of microwave irradiation.. J Phys Chem B.

[13] Hardman R (2006). A toxicologic review of quantum dots: toxicity depends on physicochemical and environmental factors.. Environ Health Persp.

[14] Lovrić J, Cho SJ, Winnik FM, Maysinger D (2005). Unmodified cadmium telluride quantum dots induce reactive oxygen species formation leading to multiple organelle damage and cell death.. Chem Biol.

[15] Schneider R (2009). The exposure of bacteria to CdTe-core quantum dots: the importance of surface chemistry on cytotoxicity.. Nanotechnology.

[16] Gaponik N, Talapin DV, Rogach AL, Hoppe K, Shevchenko EV (2002). Thiol-capping of CdTe nanocrystals: an alternative to organometallic synthetic routes.. J Phys Chem.

[17] Tian J, Liu R, Zhao Y, Xu Q, Zhao S (2009). Controllable synthesis and cell-imaging studies on CdTe quantum dots together capped by glutathione and thioglycolic acid.. J Colloid Interface Sci.

[18] Zheng Y, Gao S, Ying J (2007). Synthesis and cell-imaging applications of glutathione-capped CdTe quantum dots.. Adv Mater.

[19] Ying E, Li D, Guo S, Dong S, Wang J (2008). Synthesis and bio-imaging application of highly luminescent mercaptosuccinic acid-coated CdTe nanocrystals.. PLoS ONE.

[20] Chan WC, Nie S (1998). Quantum dot bioconjugates for ultrasensitive nonisotopic detection.. Science.

[21] Wolcott A, Gerion D, Visconte M, Sun J, Schwartzberg A (2006). Silica-coated CdTe quantum dots functionalized with thiols for bioconjugation to IgG proteins.. J Phys Chem.

[22] Wang J, Long Y, Zhang Y, Zhong X, Zhu L (2009). Preparation of highly luminescent CdTe/CdS core/shell quantum dots.. Chem Phys Chem.

[23] Peng ZA, Peng X (2001). Formation of high-quality CdTe, CdSe, and CdS nanocrystals using CdO as precursor.. J Am Chem Soc.

[24] Schüler D (2008). Genetics and cell biology of magnetosome formation in magnetotactic bacteria.. FEMS Microbiol Rev.

[25] Ahmad A, Senapati S, Khan MI, Kumar R, Ramani R (2003). Intracellular synthesis of gold nanoparticles by a novel alkalotolerant actinomycete, *Rhodococcus* species.. Nanotechnology.

[26] Samadi N, Golkaran D, Eslamifar A, Jamalifar H, Fazeli MR (2009). Intra/extracellular biosynthesis of silver nanoparticles by an autochthonous strain of *Proteus mirabilis* isolated from photographic waste.. J Biomed Nanotechnol.

[27] Bao H, Hao N, Yang Y, Zhao D (2010). Biosynthesis of biocompatible cadmium telluride quantum dots using yeast cells.. Nano Res.

[28] Bao H, Lu Z, Cui X, Qiao Y, Guo J (2010). Extracellular microbial synthesis of biocompatible CdTe quantum dots.. Acta Biomater.

[29] Calderón IL, Arenas FA, Pérez JM, Fuentes DE, Araya MA (2006). Catalases are NAD(P)H-dependent tellurite reductases.. PLoS ONE.

[30] Pérez JM, Pradenas GA, Navarro CA, Henríquez DR, Pichuantes SE (2006). *Geobacillus stearothermophilus* LV *cadA* gene mediates resistance to cadmium, lead and zinc in *zntA* mutants of *Salmonella enterica* serovar Typhimurium.. Biol Res.

[31] Pérez JM, Calderón IL, Arenas FA, Fuentes DE, Pradenas GA (2007). Bacterial toxicity of potassium tellurite: unveiling an ancient enigma.. PLoS ONE.

[32] Chasteen TG, Fuentes DE, Tantaleán JC, Vásquez CC (2009). Tellurite: history, oxidative stress, and molecular mechanisms of resistance.. FEMS Microbiol Rev.

[33] Helbig K, Bleuel C, Krauss GJ, Nies DH (2008). Glutathione and transition-metal homeostasis in *Escherichia coli*.. J Bacteriol.

[34] Turner RJ, Weiner JH, Taylor DE (1999). Tellurite-mediated thiol oxidation in *Escherichia coli*.. Microbiology.

[35] Schafer FQ, Buettner GR (2001). Redox environment of the cell as viewed through the redox state of the glutathione disulfide/glutathione couple.. Free Radic Biol Med.

[36] Mao W, Guo J, Yang W, Wang C, He J (2007). Synthesis of high-quality near-infrared-emitting CdTeS alloyed quantum dots via the hydrothermal method.. Nanotechnology.

[37] Zhang L, Xu C, Li B (2009). Simple and sensitive detection method for chromium (VI) in water using glutathione-capped CdTe quantum dots as fluorescent probes.. Microchim Acta.

[38] Eom KD, Kim JS, Park SM, Kim MS, Yu R (2006). A facile synthesis and physical properties of nano-sized dendritic alpha, epsilon-poly(L-lysine)s for the delivery of nucleic acids.. J Nanosci Nanotechnol.

[39] Zhang W, Chen G, Wang J, Ye BC, Zhong X (2009). Design and synthesis of highly luminescent near-infrared-emitting water-soluble CdTe/CdSe/ZnS core/shell/shell quantum dots.. Inorg Chem.

[40] Yang Q, Tang K, Wang F, Wang C, Qian Y (2003). A γ-irradiation reduction route to nanocrystalline CdE (E = Se, Te) at room temperature.. Materials Lett.

[41] Martin RB, Edsall JT (1958). Glutathione: ionization in basic solutions and molecular rearrangement in strongly acid solutions.. Bull Soc Chim Biol (Paris).

[42] Lovrić J, Bazzi HS, Cuie Y, Fortin GR, Winnik FM (2005). Differences in subcellular distribution and toxicity of green and red emitting CdTe quantum dots.. J Mol Med.

[43] Wu C, Shi L, Li Q, Jiang H, Selke M (2010). Probing the dynamic effect of cys-CdTe quantum dots toward cancer cells in vitro.. Chem Res Toxicol.

[44] Villena J, Henriquez M, Torres V, Moraga F, Díaz-Elizondo J (2008). Ceramide-induced formation of ROS and ATP depletion trigger necrosis in lymphoid cells.. Free Radic Biol Med.

[45] Hetz CA, Hunn M, Rojas P, Torres V, Leyton L (2002). Caspase-dependent initiation of apoptosis and necrosis by the Fas receptor in lymphoid cells: onset of necrosis is associated with delayed ceramide increase.. J Cell Sci.

[46] Dumas EM, Ozenne V, Mielke RE, Nadeau JL (2009). Toxicity of CdTe quantum dots in bacterial strains.. Transac Nanobiosci.

[47] Lewinski N, Colvin V, Drezek R (2008). Cytotoxicity of nanoparticles.. Small.

[48] Braydich-Stolle L, Hussain S, Schlager JJ, Hofmann MC (2005). In vitro cytotoxicity of nanoparticles in mammalian germline stem cells.. Toxicol Sci.

[49] Conroy J, Byrne SJ, Gun'ko YK, Rakovich YP, Donegan JF (2008). CdTe nanoparticles display tropism to core histones and histone-rich cell organelles.. Small.

[50] Demas JN, Crosby GA (1971). The measurement of photoluminescence quantum yields. A review.. J Phys Chem.

[51] Valenzuela M, Pérez-Pérez G, Corvalán AH, Carrasco G, Urra H (2010). *Helicobacter pylori*-induced loss of the inhibitor-of-apoptosis protein survivin is linked to gastritis and death of human gastric cells.. J Infect Dis.

