# Supplementary figures and images for: Biomimetic, Mild Chemical Synthesis of CdTe-GSH Quantum Dots with Improved Biocompatibility
Source: PLoS One. 2012 Jan 23;7(1):e30741. doi: 10.1371/journal.pone.0030741 (PMC3264638; doi:10.1371/journal.pone.0030741)

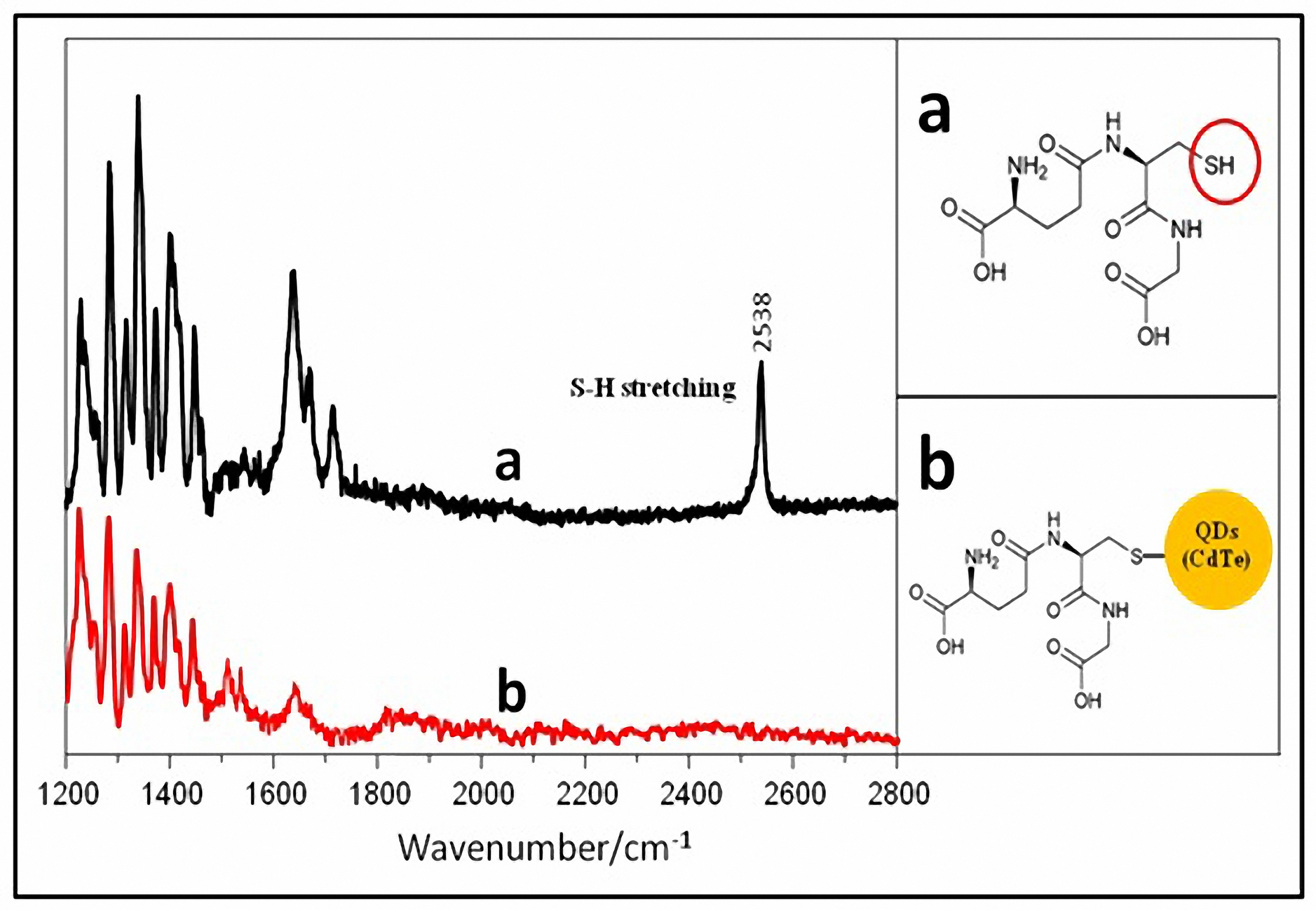

Supplement: Figure S1 — Raman vibrational spectra of GSH (a) and CdTe-GSH QDs (b). (TIF) [file pone.0030741.s001.tif]

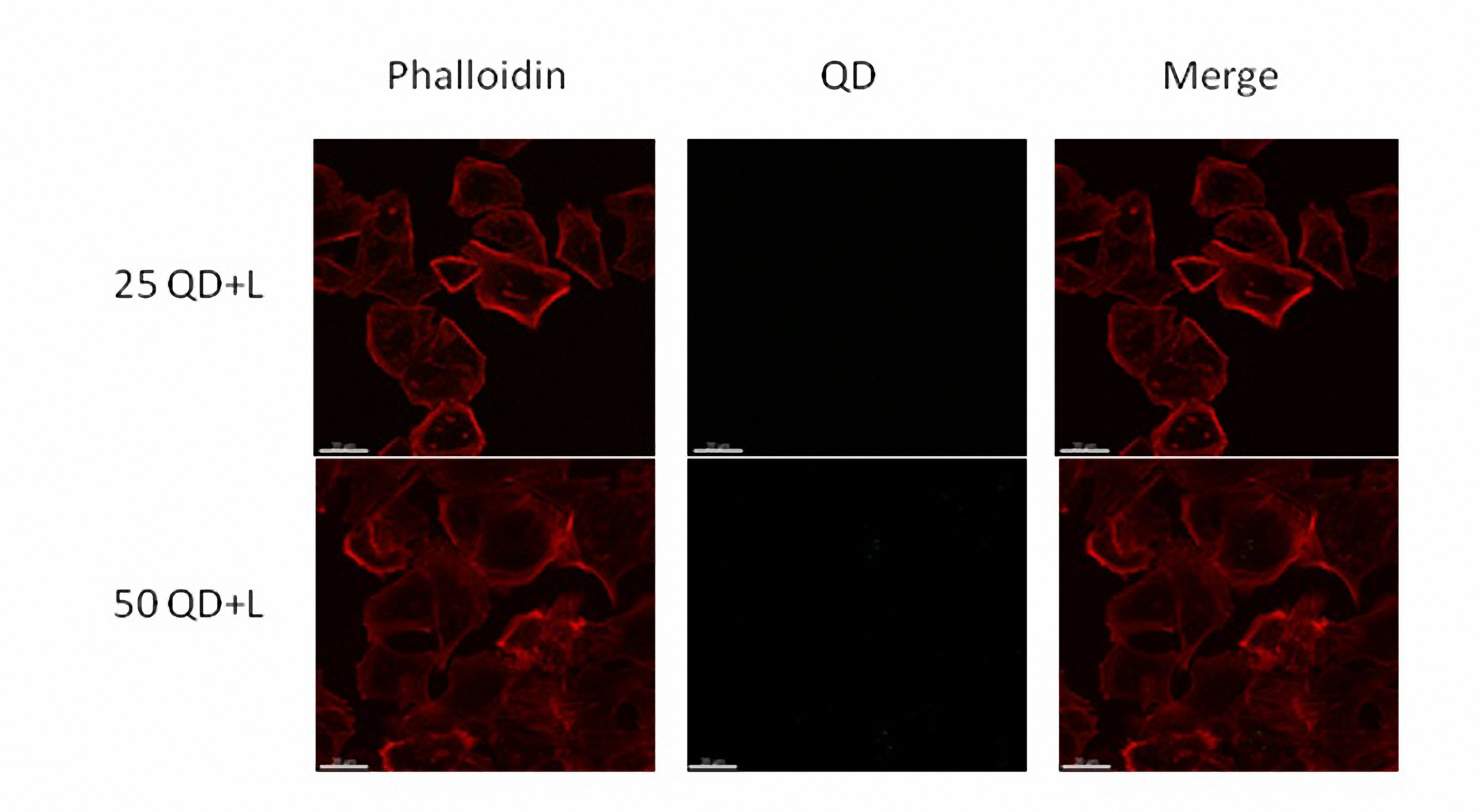

Supplement: Figure S2 — Confocal fluorescence images of MKN45 cells incubated with 25 or 50 µg/ml CdTe-GSH quantum dots in the presence of lipofectamine (25 QD+L or 50 QD+L). QDs are shown in green and cell cytoplasm was stained by phalloidin (red). (TIF) [file pone.0030741.s002.tif]

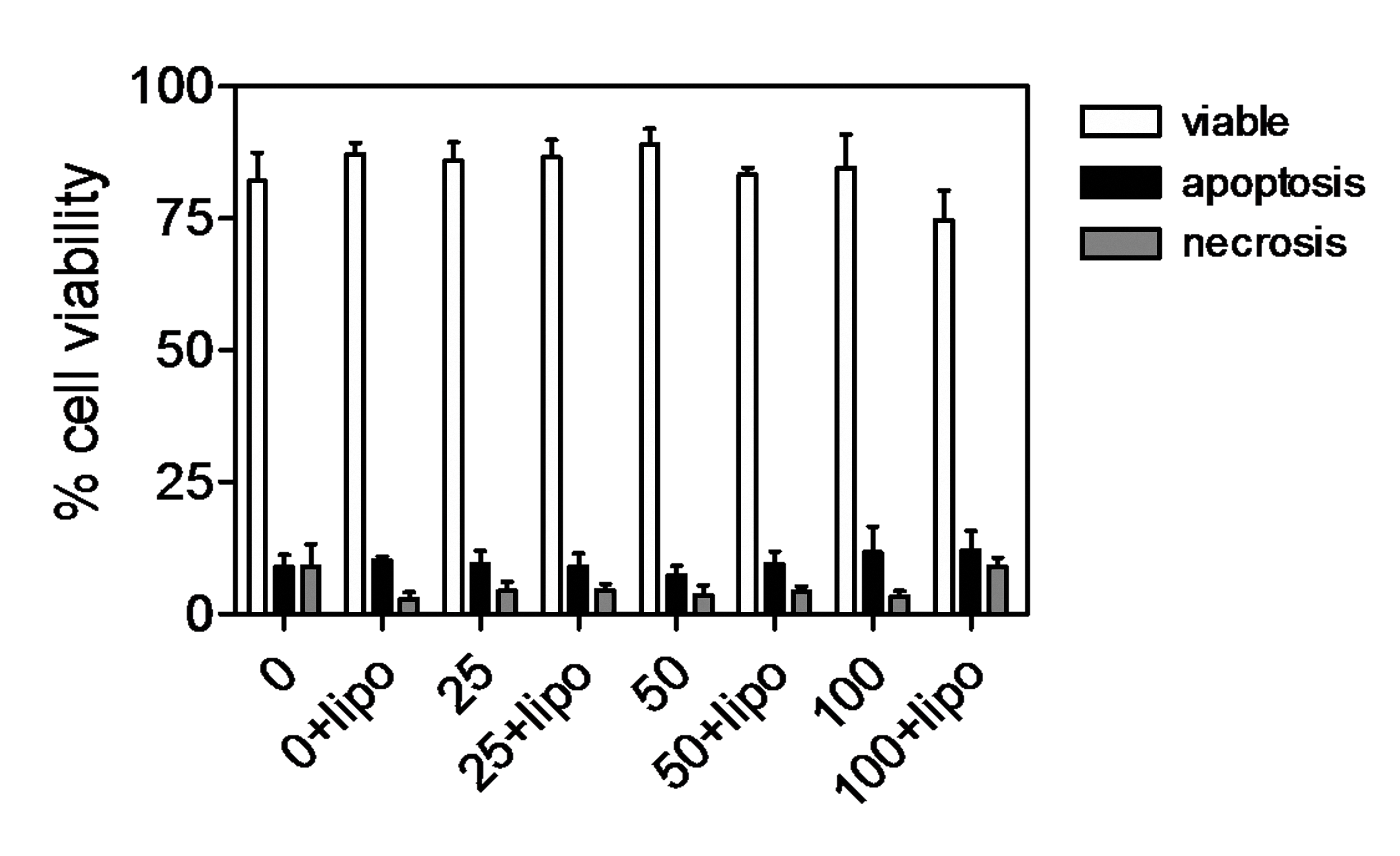

Supplement: Figure S3 — Characterization of cell death in MKN45 cells incubated with QDs at the indicated concentrations (mg/ml), with or without lipofectamine. (TIF) [file pone.0030741.s003.tif]
