# Supplementary material for: Biomimetic, Mild Chemical Synthesis of CdTe-GSH Quantum Dots with Improved Biocompatibility
Source: PLoS One. 2012 Jan 23;7(1):e30741. doi: 10.1371/journal.pone.0030741 (PMC3264638; doi:10.1371/journal.pone.0030741)
Supplement: Table S1 — Cd and Te content in the as-prepared NPs as determined by ICP. (DOCX) [file pone.0030741.s004.docx]

Table S1. Cd and Te content in the as-prepared NPs as determined by ICP.

| NPs | **μg Te/mg*** | **μg Cd/mg** | **Cd/Te ratio** |
| --- | --- | --- | --- |
| Green | 4.3 | 9.8 | 2.3 |
| Yellow | 3.0 | 9.1 | 3.0 |
| Red | 2.1 | 11.9 | 5.8 |

* element/mg sample
